# (2-Aminobenzothiazole)-Methyl-1,1-Bisphosphonic Acids: Targeting Matrix Metalloproteinase 13 Inhibition to the Bone

**DOI:** 10.3390/ph14020085

**Published:** 2021-01-24

**Authors:** Antonio Laghezza, Luca Piemontese, Leonardo Brunetti, Alessia Caradonna, Mariangela Agamennone, Fulvio Loiodice, Paolo Tortorella

**Affiliations:** 1 Department of Pharmacy and Pharmaceutical Sciences, University of Bari “A. Moro”, via E. Orabona 4, 70125 Bari, Italy; antonio.laghezza@uniba.it (A.L.); luca.piemontese@uniba.it (L.P.); leonardo.brunetti@uniba.it (L.B.); a.caradonna@hotmail.it (A.C.); 2 Department of Pharmacy, University “G. d’Annunzio” of Chieti-Pescara, Via Dei Vestini, 31, 66100 Chieti, Italy; magamennone@unich.it

**Keywords:** Matrix Metalloproteinase inhibitors, bone targeting, bisphosphonates, antitumor agents, skeletal malignancies

## Abstract

Matrix Metalloproteinases (MMPs) are a family of secreted and membrane-bound enzymes, of which 24 isoforms are known in humans. These enzymes degrade the proteins of the extracellular matrix and play a role of utmost importance in the physiological remodeling of all tissues. However, certain MMPs, such as MMP-2, -9, and -13, can be overexpressed in pathological states, including cancer and metastasis. Consequently, the development of MMP inhibitors (MMPIs) has been explored for a long time as a strategy to prevent and hinder metastatic growth, but the important side effects linked to promiscuous inhibition of MMPs prevented the clinical use of MMPIs. Therefore, several strategies were proposed to improve the therapeutic profile of this pharmaceutical class, including improved selectivity toward specific MMP isoforms and targeting of specific organs and tissues. Combining both approaches, we conducted the synthesis and preliminary biological evaluation of a series of (2-aminobenzothiazole)-methyl-1,1-bisphosphonic acids active as selective inhibitors of MMP-13 via in vitro and in silico studies, which could prove useful for the treatment of bone metastases thanks to the bone-targeting capabilities granted by the bisphosphonic acid group.

## 1. Introduction

The remodeling of the extracellular matrix (ECM) is an essential process for the development and maintenance of numerous organs and tissues, such as bone. This process is regulated by a variety of mediators and enzymes, among which Matrix Metalloproteinases (MMPs) are widely considered to be the most important players [1,2,3]. Their expression is finely regulated, and their activity is also modulated by tissue inhibitors of MMPs (TIMPs) [3].

In bone, MMPs are physiologically involved in several processes, including cellular differentiation of osteoblasts, bone formation, bone resorption, and osteoclast recruitment and migration. During the metastatic process, MMPs are involved in the so-called “vicious cycle” established by malignant cells. In order to carve out the metastatic niche, these cells trigger RANK-Ligand (RANKL) production by osteoblasts, which promotes osteoclast differentiation and bone matrix resorption; the degradation of the extracellular matrix results in the release of growth factors, driving forward tumor growth and fueling the vicious cycle [4,5,6].

Bone metastases are linked to so-called skeletal-related events, which include spinal cord compression and bone fractures. Moreover, they cause chronic pain and bone marrow aplasia and impair patient mobility, resulting in a severe reduction in quality of life. A wide range of treatments is available for bone metastasis (e.g., radiotherapy, chemotherapy, endocrine treatments, and orthopedic intervention). In this context, bisphosphonates, such as zoledronic acid, were standard-of-care for more than a decade and currently share the spotlight with RANKL-targeted monoclonal antibodies, such as Denosumab, for the treatment of this condition. Regrettably, although these therapeutic options significantly reduce the morbidity related to bone metastases, they are generally palliative and do not lead to tumor eradication [7,8,9,10].

Likewise, the ongoing research efforts toward the development of Matrix Metalloproteinase Inhibitors (MMPIs) have not yet resulted in satisfying clinical applications, mostly due to a lack of selectivity caused by the hydroxamic zinc-binding group (ZBG), typical of the first generation of MMPIs. Indeed, indiscriminate inhibition of MMPs and other zinc proteases leads to severe adverse reactions, ranging from pain to musculoskeletal syndromes, since zinc ions are essential to an enormous variety of biological processes [1,11]. Therefore, in the last decade, most research efforts focused on curbing the adverse effects of these compounds by acting on their selectivity or by targeting them to specific tissues, such as bone. This was achieved either by designing nonzinc-binding MMPIs, which target allosteric sites, or by developing compounds containing novel, more selective ZBGs [1,3,12].

Bisphosphonic acids belong to the latter class of compounds due to the bone-seeking nature of the bisphosphonic moiety itself. In the last few years, they proved to be a reliable and versatile scaffold for the development of novel bone-seeking MMPIs [13,14,15,16]. Lead compound ML115 showed high potency and was also endowed with antiosteoclastic activity, which could reduce skeletal disease burden in patients with conditions involving abnormal bone resorption [8,17]. Further developments along this line of research showed that modifications on the arylbisphosphonic scaffold could afford compounds which selectively inhibit MMP-2 [8,15,17] and MMP-9 [18], and that also exhibit significant potential for bone malignancy therapy, being superior at promoting cancer apoptosis than standard-of-care bisphosphonates, such as zoledronic acid [8,17]. Moreover, these bisphosphonic MMPIs (BMMPIs) showed no particular side effects in vivo at therapeutic dosages [8].

The significant inhibitory potency of ML115 toward MMP-2 and MMP-8 can be explained by the increased hydrophobic interactions that the biphenyl group forms with the deep S1′ sites of such MMP isoforms [19]. However, inhibition of MMP-13, an enzyme with an equally deep S1′ pocket [20], has not yet been investigated as part of the activity spectrum of bisphosphonic acids. This MMP isoform is strongly overexpressed in the metastatic microenvironment, where it plays a central role by activating MMP-9 and osteolysis, resulting in the release of growth factors that further stimulate cancer cell proliferation. Indeed, in recent years, various studies validated MMP-13 as a therapeutic target for the treatment of bone metastases [21,22,23,24]. Following this particular line of research, we report the development of a series of (2-aminobenzothiazole)methyl-1,1-bisphosphonic acids with particular attention to their activity as MMP-13 inhibitors, and provide a rationalization of their activity profile via computational studies.

## 2. Results and Discussion

Compounds **1**–**12** (reported in Table 1) were specifically developed to evaluate how the substitution of the biphenylsulfonamide of ML115 with a 2-aminobenzothiazole moiety could modify the MMP inhibition profile of these compounds, and whether substitutions on the benzothiazole scaffold could be used to further modulate their activity.

**Figure 1:**
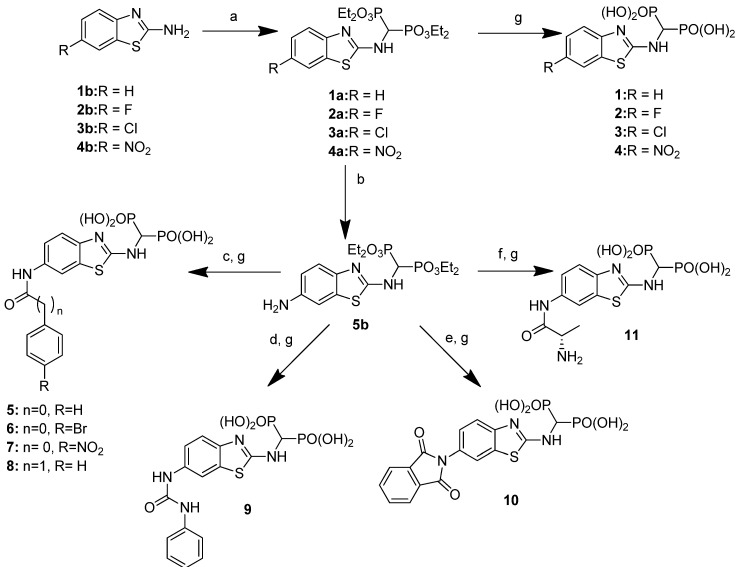
Synthesis of compounds **1–11**. Reagents and conditions: (**a**) HC(OEt)_3_, HP(OEt)_2_, 160°C, 6 h (15–53%); (**b**) H_2_, Pd/C 10%, EtOH, 3 bar, rt, 12 h (77%); (**c**) RCOOCl, NEt_3_, THF dry, 0 °C → rt, 3–12 h (82%); (**d**) phenyl isocyanate, toluene dry, reflux, 2 h (57%); (**e**) phthalic anhydride, glacial AcOH, reflux, 4 h (54%); (**f**) EDC, DMAP, Boc-L-Ala, CHCl_3_, rt, 12 h (47%); (**g**) TMBS, CH_2_Cl_2_ dry, rt, 24–48 h (40–96%).

Treatment of the appropriate commercially available 2-aminobenzothiazole with triethyl orthoformate and diethyl phosphite afforded tetraethyl-1,1-bisphosphonates 1a–4a. These bisphosphonic esters were dealkylated with trimethylsilyl bromide (TMBS) in anhydrous acetonitrile, affording bisphosphonic acids 1–4. Intermediate 4a, bearing a nitro group, was also separately reduced to compound **5b**, which was the key to the synthesis of compounds **5–11**. For compounds **5–8**, the precursor was acylated with the appropriate acyl chloride and subsequently dealkylated with TMBS. Compounds **9–11** were instead obtained via condensation with phenyl isocyanate, phthalic anhydride or Boc-L-Ala and subsequent dealkylation of the bisphosphonic ester intermediates with TMBS (Figure 1).

**Figure 2:**
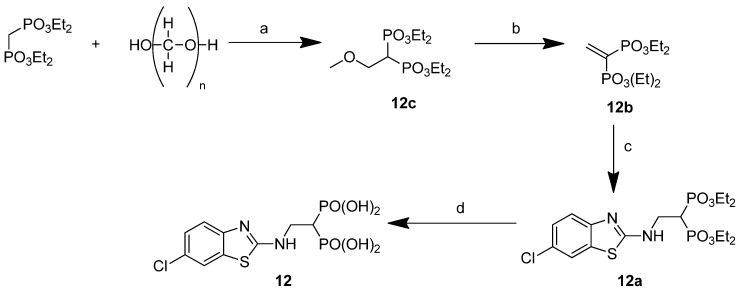
Synthesis of compound **12**. Reagents and conditions: (**a**) diethylamine, MeOH, reflux 24 h, (**b**) *p*-TsA, toluene, reflux, 6 h (yield a, b 81%); (**c**) 2-amino-6-chlorobenzothiazole, CHCl_3_ dry, N_2_, 40 °C, 27 h (65%); (**d**) 3N HCl, reflux, 17 h (96%).

Compound **12** required a different synthetic route, involving the preparation of a tetraethyl ethenyliden-bisphosphonate (**12b**), which was condensed with 2-amino-6-chlorobenzothiazole, affording intermediate **12a**, whose dealkylation in acidic conditions resulted in the desired product **12** (Figure 2).

Compounds **1**–**12** were evaluated in an enzyme inhibition assay against MMP-2, -8, -9, and -13, whose results are reported in Table 2, with ML 115 as a reference compound.

At a first glance, the most apparent characteristic of these compounds, when compared to ML115, was reduced activity toward MMP-2 and MMP-8. Interestingly, such a decrease occurred more markedly for MMP-8, which is a known antitarget in the treatment of metastasis [1,3]. Our results show that adding a substituent in position 6 of the benzothiazole ring offered the opportunity to improve inhibition of MMP-2. The most potent compounds were **5** and **7**, with IC_50_ values of 1.16 µM and 0.98 µM, respectively; these two showed to be among the most selective toward MMP-8 (7.76 and 6.84, respectively).

Compared to ML-115, our novel benzothiazole derivatives gained activity against MMP-9 influenced by the steric and electronic effects of the various substituents. Starting from analogues **1–7**, an increase in activity was observed as the substituents became bulkier and more electron-withdrawing, with the notable exception of compound **6**, whose potency dropped sharply.

The most significant result of these compounds was, however, their activity as inhibitors of MMP-13. This was not surprising, seeing as, along with MMP-2 and MMP-8, this enzyme isoform presents a deep S1′ specificity pocket [20]. Indeed, the length of the substituent seems to be a crucial parameter for the activity of these compounds; shorter compounds, such as **1–4,** were two to ten times less potent than longer derivatives **5** and **7**, which instead showed the optimal length for MMP-13 inhibition. Compound **11**, with an (L)-Ala residue, showed a stark decrease in potency, probably due to the polarity of the free amino group and the more flexible nature of this substituent. Even longer substituents, as seen with compounds **6**, **8**, and **9**, or more rigid ones (**10**), also led to a marked loss of potency.

Compound **12** showed an interesting behavior. With respect to its homologue **3**, the longer spacer between the BP group and the aryl portion improved activity toward MMP-13 while reducing potency toward MMP-2, -8, and -9 with optimal Ligand Efficiency (LE) and selectivity (especially toward MMP-8, as seen in Table 3).

The higher selectivity of these compounds toward MMP-13 is not unwelcome for the development of novel BMMPIs. Indeed, MMP-13 favors the formation of metastasis by modeling the premetastatic niche and stimulating the processes of angiogenesis, thereby resulting in enhanced survival and growth of cancer cells in their metastatic environment [22]. Furthermore, MMP-13 is highly expressed in lung and prostate malignancies [1,3], is a validated therapeutic target for a variety of pathologies that imply abnormal tissue degradation [21], and is not involved in the adverse effects of broad-spectrum MMPIs. Selective inhibitors of MMP-13 were developed over the years that, instead of binding the zinc ion, exploit the interaction with an accessory pocket named S1′* [3,25]. Although the latest research efforts in the field of MMP-13 inhibitors focused on the development of nonzinc-binding ligands, a recent approach, involving the introduction of a zinc-binding group to an otherwise nonzinc-binding class of compounds, yielded compounds capable of selectively inhibiting this enzyme in the submicromolar range [26].

Docking studies confirmed that these compounds inhibit MMPs thanks to both their ZBG (the bisphosphonic function) and their aromatic moiety that interacts with the S1′ site, a hydrophobic pocket that influences ligand selectivity of MMP inhibitors.

The main difference between ML115 and the benzothiazole series is the lack of the sulfonamide function. This group is known to be a key H-bond acceptor that, by binding to NH of Leu164 and Ala165 (MMP-13 numbering), addresses the aromatic portion in the S1′ site of MMPs [27].

The absence of the sulfonamide moiety causes the linker between the bisphosphonate and the aromatic portion to be one atom shorter, making the contemporary binding of both functions more difficult and accounting for the lower potency measured for this class of ligand with respect to sulfonamide bisphosphonates.

For this reason, moreover, the binding mode obtained for these ligands in all MMPs is not well conserved; one phosphonic group coordinates the zinc ion following the crystallographic tetrahedral geometry, while the other can line up to the binding mode observed in the X-ray complex [18] for some ligands. At the same time, for other ligands, the second phosphonic group reaches Leu164 and Ala165 NHs, providing key H-bonds and allowing the aromatic group to reach deeper in the S1′ site, as observed for arylamino-derivatives that possess the same structural frame [15]. The alpha NH often forms a H-bond with the Ala165 CO.

The better activity observed for these molecules toward MMP-13 can be attributed to the larger flexibility of the S1′ loop of this isoform with respect to other MMPs [28], giving relief to the conformational strain paid by ligands to adapt to the binding site, even if the observed interactions in the docked poses were almost the same.

**Figure 3:**
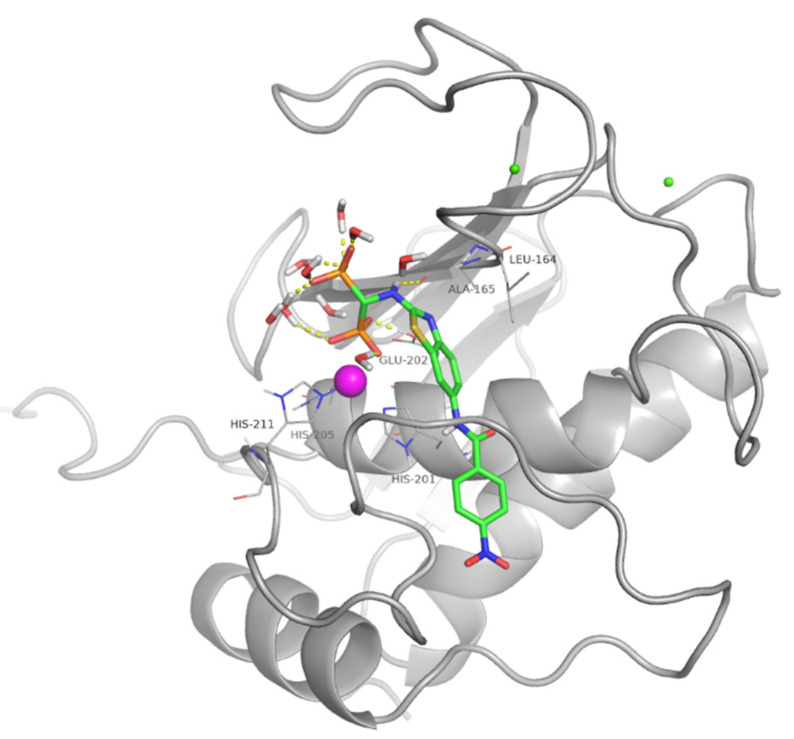
Docked pose of compound **7** (stick, green C atoms) in the MMP-13 binding site (grey cartoon). Most relevant residues are represented as lines and H-bonds are represented by yellow dashed lined.

The binding of compound **7**, the most active inhibitor of MMP-13 in the series, to the binding site of this enzyme is reported as an example (Figure 3).

In order to further validate the obtained binding geometries, additional docking calculations were carried out for the studied ligands using Autodock 4.2 [29]. Applied methods and results are reported in the Supplementary Material, and the similarity of the poses obtained with both programs is shown, once again using compound **7** as an example, in Figure S1.

## 3. Materials and Methods

### 3.1. MMP Inhibition Assays

Catalytic domains of MMP-2, -8, -9, and -13 were obtained from Enzo Life Sciences. 96-well white microtiter plates (Corning, NBS) were used to carry out the assays (in triplicate). The assay measurements were performed by preparing dilutions to six different concentrations (1 nM–100 µM) of each inhibitor in a fluorometric assay buffer (50 mM Tris·HCl pH 7.5, 200 mM NaCl, 1 mM CaCl_2_, 1 µM ZnCl_2_, 0.05% Brij-35, and 1% DMSO). Incubation of the enzyme and inhibitor solutions occurred for 15 min at room temperature; fluorogenic substrate solution (OmniMMP^®^ = Mca-Pro-Leu-Gly-Leu-Dpa-Ala-Arg-NH_2_, Enzo Life Sciences, 2.5 µM final concentration or OmniMMP^®^RED = TQ3-GABA-Pro-Cha-Abu-Smc-His-Ala-Dab (6′-TAMRA)-Ala-Lys-NH_2_, Enzo Life Sciences, and 1 µM final concentration) was subsequently added. The assay was incubated for 2–4 h at 37 °C, after which a Perkin–Elmer Victor V3 plate reader was used to measure fluorescence (λex= 340 nm, λem = 405 nm or λex = 545 nm, λem = 572 nm). Included in the assay were control wells lacking any inhibitor. MMP activity was thus determined and expressed in relative fluorescence units (RFU). Percent inhibition was calculated from control wells and IC_50_ values were determined using GraphPad Prism 5.0 and are shown as mean ± SEM of at least three independent measurements, which were performed in triplicate.

### 3.2. Chemical Methods

All reagents were purchased from Sigma Aldrich Chemicals (Milan, Italy) and were used without purification. The reactions were monitored by TLC (silica gel, UV_254_) with UV light (short wave ultraviolet 254 nm and long wave ultraviolet 365 nm). All anhydrous reactions were performed under argon or nitrogen atmosphere. The column chromatography was performed using Fluka silica gel 60 Ȧ (63–200 µm) or silica gel Si 60 (40–63 µm). Mass spectra were recorded on an HP MS 6890-5973 MDS spectrometer, electron impact 70 eV, equipped with an HP ChemStation or with an Agilent 6530 Series Accurate-Mass Quadrupole Time-of-FLIGHT (Q-TOF) LC/MS. High-resolution mass spectrometry (HRMS) analyses were performed using a Bruker microTOF QII mass spectrometer equipped with an electrospray ion source (ESI). ^1^H NMR was recorded using the suitable deuterated solvent on a Varian Mercury 300 or 500 NMR Spectrometer. Chemical shift (δ) was expressed as parts per million (ppm) and the coupling constant (J) in Hertz (Hz). Melting points were determined in open capillaries on a Gallenkamp electrothermal apparatus and were uncorrected.

#### 3.2.1. General Procedure for the Preparation of Tetraethyl Bisphosphonates (**1a**–**4a**)

Triethyl orthoformate, diethyl phosphite and the opportune 2-aminobenzothiazole were added to a round-bottom flask fitted with a distillation apparatus in a 1.2:3:1 stoichiometric ratio. The resulting mixture was then heated to 160 °C until all EtOH was distilled away; the residue was dissolved in ethyl acetate, and evaporated away under vacuum, affording a crude yellow oil, which was further purified via column chromatography over silica gel (eluent EtOAc/MeOH 9:1). The desired compounds were obtained as white solids in 15–53% yields.

**Tetraethyl [(benzo[*d*]thiazol-2-amino)methyl]-1,1-bisphosphonate (1a):** White solid, 42% yield. 1H NMR (500 MHz, CDCl_3_): δ = 1.26–1.32 (m, 12H, CH_3_), 4.17–4.30 (m, 8H, CH_2_), 5.26 (t, J_HP_ = 21.52, 1H, PCHP), 6.17 (bs, 1H, NH), 7.12 (t, J = 7.83,1H, aromatic), 7.30 (t, J = 7.83,1H, aromatic), 7.55–7.59 (m, 2H, aromatics). MS (ESI): *m*/*z*: 437[M+H]+; MS2 *m*/*z* (%): 299(46), 271 (88), 243(100), 225(66), 161 (39).

**Tetraethyl [(6-fluorobenzo[d]thiazol-2-amino)methyl]-1,1-bisphosphonate (2a):** White solid, 53% yield. 1H NMR (500 MHz, CDCl_3_): δ = 1.26–1.31 (m, 12H, CH_3_), 4.18–4.27 (m, 8H, CH_2_), 5.19 (t, 1H, PCHP), 5.95 (bs, 1H, NH), 7.00–7.03 (m, 1H, aromatic), 7.26–7.29 (m, 1H, aromatic), 7.45–7.48 (m, 1H, aromatic). MS(ESI): *m*/*z*: 455 [M+H]+; MS2: *m*/*z* (%): 317(38), 289(89), 261(100), 243 (75), 179(39).

**Tetraethyl [(6-chlorobenzo[*d*]thiazol-2-amino)methyl]-1,1-bisphosphonate (3a):** White solid, 15% yield. 1H NMR(500 MHz, CDCl_3_): δ = 1.26–1.31 (m, 12 H, CH_3_), 4.16–4.29 (m, 8H, CH_2_), 5.19 (t, J_HP_ = 21.53, 1H, PCHP), 5.80 (bs, 1H, NH), 7.24–7.27 (dd, J_1_ = 8.81, J_2_ = 2.45, 1H, aromatic), 7.44 (d, J = 8.81, 1H, aromatic), 7.54 (d, J = 2.45, 1H, aromatic). MS(ESI): *m*/*z*: 495 [M+2+Na]+, 493[M+Na]+, 473[M+2+H]+, 471[M+H]+; MS2(471): *m*/*z* (%): 335 (27), 333(43), 307(49), 305(86), 279 (45), 277(100), 197 (18), 195 (41).

**Tetraethyl [(6-nitrobenzo[d]thiazol-2-amino)methyl]-1,1-bisphosphonate (4a):** Yellow solid, 40% yield. 1H NMR(500 MHz, CDCl3): δ = 1.26 (t, J = 7.095, 6H, CH3), 1.33 (t, J = 7.095, 6H, CH3), 1.7(bs, 1H, NH), 4.18–4.32 (m, 8H, CH_2_), 5.29 (t, J_HP_ = 21.53, 1H, PCHP), 7.55 (d, J = 8.81, 1H, aromatic), 8.19–8.21(dd, J_1_ = 8.81, J_2_ = 2.45, 1H, aromatic), 8.48 (d, J = 2.45, 1H, aromatic). MS(ESI): *m*/*z*: 482[M+H]+; MS2: *m*/*z* (%): 344(18), 316.01(69), 288 (100), 272 (12), 206 (25), 139 (16).

**Tetraethyl [(6-aminobenzo[d]thiazol-2-amino)methyl]-1,1-bisphosphonate (5b):** Ten percent Pd/C (0.20 mmol) was added to the solution of the nitro compound (0.532 mmol) in 8.5 mL EtOH, and the mixture was hydrogenated at room temperature at a pressure of 3 bar for 12 h. The reaction mixture was filtered through a pad of celite and the filtrate was evaporated in vacuo to give an oil. The residue was purified by chromatography on silica gel (eluent: CHCl_3_/ MeOH 9.5: 0.5 *v*/*v*) to give the desired amino derivate. White solid, 77% yield. 1H NMR (CDCl_3_, 500 MHz): δ = 1.25–1.30 (m, 12H, CH_3_), 3.64 (b, 2H, NH_2_), 4.09–4.27 (m, 8H, CH_2_), 5.15 (t, J_HP_ = 21.53, 1H, PCHP), 5.52 (bs, 1H, NH), 6.67 (dd, J_1_ = 8.32, J_2_ = 2.45, 1H, aromatic), 6.91 (d, J = 2.45, 1H, aromatic), 7.35 (d, J = 8.32, 1H, aromatic). MS(ESI): *m*/*z*: 474[M+Na]^+^; MS_2_: *m*/*z* (%): 336 (100), 308 (73), 262 (32), 174 (24), 146 (22).

#### 3.2.2. General Procedure for N-Acylation of Compound **5b**

The appropriate acyl chloride (1.1–2 mmol) and triethylamine (2 mmol) were added to the solution of amino derivate (5b) (1 mmol) in anhydrous THF and the mixture was stirred at room temperature on nitrogen or argon for 3–12 h. After the given time, the eluent was evaporated in vacuo and the residue was partitioned between EtOAc and NaHCO_3_ and the layers were separated. The organic phase was washed with HCl 1N, NH_4_Cl ss, brine, dried over anhydrous Na_2_SO_4_, filtered and the filtrate was evaporated in vacuo. The residue was purified by chromatography on silica gel (eluent: CHCl_3_/MeOH 98:2 *v*/*v* or AcOEt /MeOH 9:1 *v*/*v*) or crystalized with AcOEt to give the desired product.

**Tetraethyl [(6-(benzamido)benzo[d]thiazol-2-amino)methyl]-1,1-bisphosphonate:** White solid, 61% yield (chromatography, eluent: CHCl_3_/MeOH 98: 2 *v*/*v*). 1H NMR (500 MHz, [D6] DMSO): δ = 1.13–1.97 (m, 12H, CH_3_), 4.06–4.12 (m, 8H, CH_2_), 5.09–5.21 (td, J_HP_ = 22.5, J_HH_ = 9.79, 1H, PCHP), 7.40 (d, J = 8.81, 1H, aromatic), 7.50–7.53 (m, 4H, aromatics), 7.92–7.94 (m, 2H, aromatics), 8.16 (d, J = 1.96, 1H, aromatic), 8.73 (d, J = 9.78, 1H, NH), 10.25 (s, 1H, NH). MS (ESI): *m*/*z*: 578 [M+Na]^+^, 556 [M+H]^+^; MS_2_(556): *m*/*z* (%): 418 (100), 390 (92), 372 (33), 381 (33), 362 (57), 344 (73), 280 (29). 554 [M-H]^−^.

**Tetraethyl [(6-(4-bromobenzamido)benzo[d]thiazol-2-amino)methyl]-1,1-bisphosphonate:** White solid, 82% yield, (AcOEt). 1H NMR(500 MHz, [D6] DMSO): δ = 1.13–1.21 (m, 12H, CH_3_), 4.07–4.10 (m, 8H, CH_2_), 5.15 (td, J_HP_ = 22.5, J_HH_ = 9.30, 1H, PCHP), 7.40 (d, J = 8.81, 1H, aromatic), 7.51 (d, J = 8.81, 1H, aromatic), 7.73 (d, J = 8.32, 2H, aromatics), 7.91(d, J = 8.32, 2H, aromatics), 8.16 (s, 1H, aromatic), 8.75 (d, J = 9.30, NH), 10.37 (s, 1H, NH). MS(ESI): *m*/*z*: 658[M+2+Na]^+^, 656[M+Na]^+^, 636[M+2+H]^+^, 634 [M+H]^+^; MS2(634): *m*/*z* (%): 498(100), 496 (70), 470 (55), 468 (62), 452 (40), 450 (32), 424 (55), 422 (47). 

**Tetraethyl [(6-(4-nitrobenzamido)benzo[d]thiazol-2-amino)methyl]-1,1-bisphosphonate:** Yellow solid, 64% yield, (AcOEt). 1H NMR (500 MHz, [D6] DMSO): δ = 1.15–1.20 (m, 12H, CH_3_), 4.07–4.12 (m, 8H, CH_2_), 5.15 (t, J_HP_ = 22.5, 1H, PCHP), 7.43 (d, J = 8.81, 1H, aromatic), 7.51–7.53 (dd, J_1_ = 8.32, J_2_ = 1.96, 1H, aromatic), 8.16–8.18 (m, 3H, aromatics), 8.68 (m, 2H, aromatics), 8.8 (bs, 1H, NH), 10.59 (s, 1H, NH). MS(ESI): *m*/*z*: 623[M+Na]^+^, 601[M+H]^+^; MS2: *m*/*z* (%): 555(24), 463 (100), 436(18.25), 389 (69), 325 (25).

**Tetraethyl [(6-(2-phenylacetamido)benzo[d]thiazol-2-amino)methyl]-1,1-bisphosphonate:** White solid, 56% yield, (AcOEt). 1H NMR (500 MHz, [D6] DMSO): δ = 1.12–1.18 (m, 12H, CH_3_), 3.60 (s, 2H, CH_2_), 3.97–4.17 (m, 8H, CH_2_), 5.13 (td, J_HP_ = 22.25, J_HH_ = 9.78, 1H, PCHP), 7.22–7.36 (m, 7H, aromatics), 8.03 (s, 1H, aromatic), 8.67 (d, J = 9.78, 1H, NH), 10.15 (s, 1H, NH). MS (ESI): *m*/*z*: 592[M+Na]^+^, 570[M+H]+; MS2 (570): *m*/*z* (%): 432(100), 404 (81), 386 (30), 358(61), 294(24). 568[M-H]^−^; MS2: *m*/*z* (%): 430(89), 339 (90), 310 (100), 287 (49), 259 (27), 190 (42), 185 (16), 137 (97), 107 (33). 

**Tetraethyl [(6-(3-phenylureido)benzo[d]thiazol-2-amino)methyl]-1,1-bisphosphonate:** A solution of phenyl isothiocyanate (1.2 mmol) in anhydrous toluene (2 mL) was added to the suspension of 5b (1 mmol) in anhydrous toluene (2 mL) and the mixture was heated to reflux for 2 h. After the given time, the eluent was evaporated in vacuo and the residue was triturated with AcOEt and filtered to afford the desired product: white solid, 57% yield. 1H NMR(300 MHz, [D6]DMSO): δ = 1.12–1.20 (m, 12H, CH_3_), 4.06–4.13 (m, 8H, CH_2_), 5.05–5.13 (td, J_HP_ = 22.26, J_HH_ = 9.96, 1H, PCHP), 6.91–9.95 (t, J = 7.02, 1H, aromatic), 7.12–7.28 (m, 3H, aromatics), 7.34 (d, J = 8.787, 1H, aromatic), 7.43 (d, J = 8.20, 2H, aromatics), 7.88 (d, J = 2.34, 1H, aromatic), 8.62 (d, J = 9.96, 1H, NH), 8.72 (s, 1H, NH), 8.75 (s, 1H, NH). MS(ESI): *m*/*z*: 593[M+Na]^+^, 571[M+H]+; MS_2_: *m*/*z* (%): 525 (17), 433 (100), 405 (77), 378 (32), 359 (58).

**Tetraethyl [(6-(1,3-dioxoisoindolin-2-yl)benzothiazol-2-amino)methyl]-1,1-bisphosphonate:** A mixture of 5b (1 mmol) and phthalic anhydride (1.07 mmol) in 6 mL of glacial acetic acid (AcOH) was refluxed for 4 h. Then, the eluent was evaporated in vacuo; the residue was diluted with EtOAc and a solution of 6 M NaOH was added until pH = 6, and the layers were separated. The organic phase was washed with brine, dried over anhydrous Na_2_SO_4_, filtered, and the filtrate was evaporated in vacuo. The residue was purified by chromatography on silica gel (eluent: CHCl_3_/MeOH 9: 1 *v*/*v*) to give the desired product: green solid, 54% yield. 1H NMR(500 MHz, [D6]DMSO): δ = 1.15–1.21 (m, 12H, CH_3_), 4.07- 4.13 (m, 8H, CH_2_), 5.14–5.25 (td, 1H, J_HP_ = 22.25, J_HH_ = 8.07, 1H, PCHP), 7.28–7.30 (dd, J_1_ = 8.33, J_2_ = 1.96, 1H, aromatic), 7.54(d, J = 8.33, 1H, aromatic), 7.76 (d, J = 1.96, 1H, aromatic), 7.88–7.97 (m, 4H, aromatics), 8.95 (d, J = 8.07, 1H, NH). MS (ESI): *m*/*z*: 604[M+Na]^+^, 582[M+H]+; MS2(582): *m*/*z* (%): 536 (23), 444 (100), 417 (23), 416 (80), 398 (30), 388 (57), 370 (72), 306 (24), 297 (11). 580[M-H]^−^; MS2: *m*/*z* (%): 137 (100), 108 (32).

**Tetraethyl [(6-((L) N-Boc 2 aminopropanamido)benzothiazol-2-amino)methyl]-1,1-bisphosphonate:** Boc-L-Ala (1 mmol), EDC (1 mmol) and DMAP (2 mmol) were added to the solution of 5b (1 mmol) in CHCl_3_ (4 mL) and the mixture was stirred at room temperature on nitrogen for 12 h. After the given time, the eluent was evaporated in vacuo, the residue was diluted with EtOAc, the organic phase was washed with brine, dried over anhydrous Na_2_SO_4_, and filtered, and the filtrate was evaporated in vacuo. The residue was triturated with AcOEt and filtered to afford the final compound: white solid, 47% yield. 1H NMR(500 MHz, [D6]DMSO): δ = 1.12–1.20 (m, 12H, CH_3_), 1.23(d, J = 6.85, 3H, CH_3_), 1.36 (s, 9H, -(CH_3_)_3_), 4.07–4.13 (m, 9H, CH_2_, CH), 5.07–5.19 (td, 1H, J_HP_ = 22.25, J_HH_ = 9.78, 1H, PCHP), 7.02 (d, J = 6.85, 1H, CH), 7.32 (dd, J_1_ = 8.32, J_2_ = 1.47, 1H, aromatic), 7.35 (d, J = 8.32, 1H, aromatic), 8.03 (d, J = 1.47, 1H, aromatic), 8.67 (d, J = 9.78, 1H, NH), 9.88 (s, 1H, NH). MS(ESI): *m*/*z*: 645[M+Na]^+^, 623[M+H]+; MS_2_ (623): *m*/*z* (%): 521(62), 430(18), 401 (19), 385 (21), 383 (25), 355 (18), 286 (12).

#### 3.2.3. General Procedure for the Preparation of 1,1-Bisphosphonic Acids (1–12)

Method A: A solution of the appropriate tetraethyl bisphosphonate (1 mmol) in 4 mL 2N HCl solution was kept at reflux for 12–24 h. After removal of the aqueous phase under reduced pressure, the crude bisphosphonic acids were triturated with the opportune solvent and filtered to afford the final compounds as white solids in 20–96% yield.

Method B: Anhydrous trimethylsilylbromide (17–32 mmol) was carefully added to a solution of the corresponding tetraethyl bisphosphonate (1 mmol) in anhydrous acetonitrile (6 mL) at 0 °C under argon and the resulting mixture was stirred at room temperature for 24–48 h. After the given time, 2 mL of MeOH was added and the mixture was stirred for 5 min. The solvent was distilled off and the crude bisphosphonic acids were triturated with the opportune solvent and filtered to afford the desired compounds as white solids in 40–100% yield.

(Benzo[d]thiazol-2-amino)methyl-1,1-bisphosphonic acid (1): Method A; white solid, 72% yield; mp: >250 °C (Et_2_O). 1H NMR (500 MHz, NaOD): δ = 2.8–4.00 (br, 6H, NH, OH, PCHP), 6.95 (t, 1H, J = 7.58, aromatic), 7.16 (t, J = 7.58, 1H, aromatic), 7.23 (d, J = 7.83, 1H, aromatic), 7.51 (d, J = 7.83, 1H, aromatic). MS(ESI): *m*/*z*: 323[M-H]^−^; MS2: *m*/*z* (%): 287(26), 241 (100), 177 (33), 149 (10). 

(6-fluorobenzo[d]thiazol-2-amino)methyl-1,1-bisphosphonic acid (2): Method A; white solid, 26% yield; mp: >250 °C (EtOAc and Acetone). 1H NMR (500 MHz, [D6] DMSO): δ = 3.67–5.45 (br, 4H, OH), 4.77 (t, J_HP_ = 21.28, 1H, PCHP), 7.06 (m, 1H, aromatic), 7.33–7.36 (m, 1H, aromatic), 7.59 (m, 1H, aromatic), 8.26–9.17 (b, 1H, NH). 31P NMR(500 MHz, [D6] DMSO): δ = 14.04 (d, JPH= 16.79, 2P, PCHP). MS (ESI): *m*/*z*: 341 [M-H]^−^; MS2: *m*/*z* (%): 305(32), 259 (100), 195 (40), 167 (13).

(6-chlorobenzo[d]thiazol-2-amino)methyl-1,1-bisphosphonic acid (3): Method A; white solid, 68% yield; mp: > 250°C (Acetone/AcOEt). 1H NMR (500 MHz, [D6] DMSO): δ = 4.00–6.00 (br, 4H, OH), 4.80 (t, J_HP_ = 20.79, 1H, PCHP), 7.23 (dd, J_1_ = 8.81, J_2_ = 1.46, 1H, aromatic), 7.34 (d, J = 8.81, 1H, aromatic), 7.77 (d, J = 1.46, 1H, aromatic), 7.86–8.26 (bs, 1H, NH). ^31^P NMR (500 MHz, [D6] DMSO): δ = 14.074 (d, 2P, PCHP). MS(ESI): *m*/*z*: 359[M+2-H]^−^, 357 [M-H]^−^; MS2: *m*/*z* (%): 323(24), 321 (41), 277 (43), 275 (100), 213 (21), 211 (44).

(6-chlorobenzo[d]thiazol-2-amino)ethyl-1,1-bisphosphonic acid (12): Method A; white solid, 96% yield; mp: > 250 °C (AcOEt). 1H NMR (500 MHz, [D6] DMSO): δ = 3.6–4.00 (br, 8H, OH, PCHP, NH, CH_2_), 7.22 (d, J = 8.56, 1H, aromatic), 7.34 (d, J = 8.56, 1H, aromatic), 7.79 (s, 1H, aromatic). ^31^P NMR (500 MHz, [D6] DMSO): δ = 17.40 (d, JPH= 22.86, 2P, PCHP). MS (ESI): *m*/*z*: 373[M+2-H]^−^, 371[M-H]^−^; MS2 *m*/*z* (%): 337(13), 335(26), 247(40), 245(100). 

(6-nitrobenzo[d]thiazol-2-amino)methyl-1,1-bisphosphonic acid (4): Method B; white solid, 76% yield; mp: >250 °C (MeOH). 1H NMR (500 MHz, [D6] DMSO): δ = 4.1–5.5 (br, 5H, OH, PCHP), 7.44 (d, J = 8.81, 1H, aromatic), 8.08 (d, J = 8.81, 1H, aromatic), 8.65 (s, 1H, aromatic), 8.65 (bs, 1H, NH). ^31^P NMR (500 MHz, [D6] DMSO): δ = 13.64 (d, J = 21.45, PCHP). MS (ESI): *m*/*z*: 368[M-H]^−^; MS2: *m*/*z* (%): 332 (45), 305 (25), 286 (58), 222 (100).

(6-benzamidobenzo[d]thiazol-2-amino)methyl-1,1-bisphosphonic acid (5): Method B; white solid, 40% yield; mp: >250 °C(Acetone/ MeOH 3:1 *v*/*v*). 1H NMR (500 MHz, [D6] DMSO): δ = 4.80 (t, J_HP_ = 20.5, 1H, PCHP), 5.00–6.4 (br, 4H, OH), 7.38 (d, J = 8.32, 1H, aromatic), 7.49–7.58 (m, 4H, aromatics), 7.93 (dd, J_1_ = 7.34, J_2_ = 1.46, 1H, aromatics), 8.16 (d, J = 1.46, 1H, aromatic), 8.95 (bs, 1H, NH), 10.24 (s, 1H, NH). 31P NMR (500 MHz, [D6] DMSO): δ = 13.69 (d, J = 16.78, PCHP). MS (ESI): *m*/*z*: 442 [M-H]^−^; MS2: *m*/*z* (%): 407 (15), 406 (62), 361 (22), 360 (100), 343 (18), 342 (69), 296 (25).

(6-(4-bromobenzamido)benzo[d]thiazol-2-amino)methyl-1,1-bisphosphonic acid (6): Method B; white solid, 78% yield; mp: >250 °C ISO). 1H NMR(500 MHz, [D6] DMSO): 2.00–3.5 (br, 6H, OH, NH, PCHP), 7.22–7.88 (m, 6H, aromatics), 8.11 (s, 1H, aromatic), 10.24 (s, 1H, NH). ^31^P NMR (500 MHz, [D6] DMSO): δ = 14.40 (d, 2P, PCHP). MS (ESI): *m*/*z*: 522[M+2-H]^−^, 520 [M-H]^−^; MS2: *m*/*z* (%): 486 (91), 484 (16), 440 (10), 438 (66), 422 (95.5), 420 (72).

(6-(4-nitrobenzamido)benzo[d]thiazol-2-amino)methyl-1,1-bisphosphonic acid (7): Method B; white solid, 96% yield; mp: > 250 °C (MeOH). 1H NMR (300 MHz, [D6] DMSO): δ = 4.73 (t, J_HP_ = 20.33, 1H, PCHP), 4.86- 6.09 (br, 5H, OH, NH), 7.45 (d, J = 7.91, 1H, aromatic), 7.63 (d, J = 7.91, 1H, aromatic), 8.17 (d, J = 8.79, 2H, aromatic), 8.29 (s, 1H, aromatic), 8.36 (d, J = 8.79, 2H, aromatics), 10.89 (s, 1H, NH). 31P NMR (500 MHz, [D6] DMSO): δ = 12.76 (d, J_PH_ = 21.24, 2P, PCHP). MS (ESI): *m*/*z*: 487[M-H]^−^; MS2: *m*/*z* (%): 452 (13), 451 (53), 406 (17), 405 (78), 388 (24), 387 (100), 341 (25), 339 (20), 233 (14).

(6-(2-phenylacetamido)benzo[d]thiazol-2-amino)methyl-1,1-bisphosphonic acid (8): Method B; white solid, 69% yield; mp: >250 °C (ISO). 1H NMR (500 MHz, [D6] DMSO): δ = 4.43 (s, 2H, CH2), 3.90–5.10 (br, 5H, OH, PCHP), 8.01–8.14 (m, 8H, aromatics), 8.81 (s, 1H, NH), 10.17 (s, 1H, NH). MS (ESI): *m*/*z*: 456[M-H]^−^; MS2: *m*/*z* (%): 420 (55), 375 (24), 374 (100), 357 (12), 356 (49), 310 (19.5).

(6-(3-phenylureido)benzo[d]thiazol-2-amino)methyl-1,1-bisphosphonic acid (9): Method B; white solid, 69% yield; mp:> 250 °C (ISO). 1H NMR (300 MHz, [D6] DMSO): δ = 4.76 (t, J_HP_ = 19.62, 1H, PCHP), 5.00–6.00 (br, 5H, OH, NH), 6.94 (t, J = 7.03, 1H, aromatic), 7.22–7.44 (m, 6H, aromatics), 7.92 (s, 1H, aromatic), 8.66 (s, 1H, NH), 8.74 (s, 1H, NH). 31P NMR (500 MHz, [D6] DMSO): 13.02 (d, 2P, PCHP). MS (ESI): *m*/*z*: 457[M-H]^−^; MS2: *m*/*z* (%): 421 (58), 376 (24), 375 (100), 357 (20), 311 (24), 238 (94).

(6-(1,3-dioxoisoindolin-2-yl)benzo[d]thiazol-2-amino)methyl-1,1-bisphosphonic acid (10): Method B; white solid, 58% yield; mp: >250 °C (ISO). 1H NMR(500 MHz, [D6] DMSO): δ = 4.20–6.00 (br, 5H, OH, PCHP), 6.97–7.25 (m, 1H, aromatic), 7.33–7.47 (m, 3H, aromatics), 7.71 (s, 1H, aromatic), 7.83–7.95 (m, 2H, aromatics), 8.7 (br, 1H, NH). 31P NMR (500 MHz, [D6] DMSO): δ = 13.02 (d, 2P, PCHP). MS (ESI): *m*/*z*: 468[M-H]^−^; MS2: *m*/*z* (%): 432 (69), 386 (100), 322 (20), 197 (18).

6-((L) 2-aminopropanamido)benzo[d]thiazol-2-amino)methyl-1,1-bisphosphonic acid (11): Method B; white solid, 94% yield; mp: >250 °C (ISO). 1H NMR (500 MHz, NaOD): δ = 1.19 (d, J = 6.85, 3H, CH3), 3.43–3.47 (q, J = 6.85, 1H, CH), 4.49–4.88 (br, 9H, OH, PCHP, NH, NH, NH2), 7.08 (dd, J_1_ = 8.80, J_2_ = 1.96, 1H, aromatic), 7.20 (d, J = 8.8, 1H, aromatic), 7.54 (d, J = 1.96, 1H, aromatic). 31P (500 MHz, NaOD): δ = 14.84 (d, J = 18.31, 2P, PCHP). MS (ESI): *m*/*z*: 409 [M-H]^−^; MS2: *m*/*z* (%): 373 (41), 327 (100), 309 (51), 263 (27).

Procedure for the preparation of Tetraethyl (2-methoxyethane-1,1-diyl)bisphosphonate (12c): Diethylamine (1 mmol) was added to the suspension of paraformaldehyde (1 mmol) in 13 mL of methanol. The system was left under reflux until complete solubilization of the suspension. Subsequently, the solution obtained was stirred at room temperature and a solution of tetraethyl methylenebisphosphonate (1.6 mmol) in 2 mL of methanol was added. The system was kept at reflux for 24 h. After the given time, the solvent was evaporated in vacuo and 3 mL of toluene was added and subsequently evaporated away three times, obtaining a yellow oil which was used in the next reaction without further purification. 1H NMR(500 MHz CDCl_3_): δ = 1.2 (t, J = 7.1, 12H, OCH_2_CH_3_), 2.52 (t, J_HP_ = 24.00, J_HH_ = 6.00, 1H, PCHP), 3.2 (s, 3H, OCH_3_), 3.63 (m, 2H, CH_3_OCH_2_), 4.02 (m, J = 7.30, 8H, OCH_2_CH_3_). GC-MS: *m*/*z* (%): 332 (0.3), 195 (100).

Procedure for the preparation of tetraethyl ethenylidenebisphosphonate (12b): p-TsA monohydrate (0.26 mmol) was added to the solution of tetraethyl (2-methoxyethyl)bisphosphonate 12c (5 mmol) in 7 mL of anhydrous toluene and the mixture was refluxed for 6 h. After the given time, the solvent was evaporated in vacuo. The residue was partitioned between CHCl_3_ and NaHCO_3_ and the layers were separated. The organic layer was washed with brine, dried over anhydrous Na_2_SO_4_, filtered, and the filtrate was evaporated in vacuo, obtaining a brown oil which was used in the next reaction without further purification: 81% yield. 1H NMR (500 MHz, CDCl3): δ = 1.34 (t, J = 7.1, 12H, CH_3_), 4.02 (m, 8H, CH_2_), 6.98 (d, 2H, C=CH_2_). GC-MS: *m*/*z* (%): 301 (3.5), 171 (100), 163 (96).

Tetraethyl (((2-(6-chlorobenzo[d]thiazol-2-yl)amino)ethane-1,1-diyl)bisphosphonate) (12a): Tetraethyl ethenylidenebisphosphonate 12b (1.2 mmol) was added to the solution of 2-amino-6-chlorobenzothiazole (1.62 mmol) in 8 mL of anhydrous CHCl_3_ and the system was stirred, under nitrogen atmosphere, at 40 °C for 27 h. After the given time, the solvent was evaporated in vacuo and the residue was purified by chromatography on silica gel (eluent: EtOAc) to give the desired product: white solid, 65% yield. 1H NMR (500 MHz, CDCl_3_): δ = 1.24–1.76 (m, 12H, CH_3_), 2.84 (tt, J_HP_ = 23, J_HH_ = 6.36, 1H, CH), 3.99–4.07 (td, J_1_ = 16.00, J_2_ = 6.36, 2H, CH_2_), 4.08–4.26 (m, 8H, CH_2_), 6.38 (bs, 1H, NH), 7.22–7.24 (dd, J_1_ = 8.81, J_2_ = 1.96, 1H, aromatic), 7.41 (d, J = 8.81, 1H, aromatic), 7.55 (d, J = 1.96, 1H, aromatic).

### 3.3. Docking Studies

All calculations were carried out using the Schrodinger Suite 2019-3 (Schrodinger, New York, NY, USA) [30]. Ligand structures were built using Maestro in a fully deprotonated form, minimized with MacroModel using the OPLS2005 force field, the PRCG algorithm, at a convergence gradient of 0.05. A Monte Carlo Multiple Minimum/Low Mode Conformational Search (MCMM/LMCS) protocol was applied for the conformational search using the automatic setup, performing 200 steps per rotatable bond. The global minimum geometry was used to follow the docking studies carried out on the four studied MMPs following the previously described procedure [13].

## 4. Conclusions

Over the years, inhibition of MMPs has been a much sought-after target for the treatment of bone metastases, showing promise in stopping the vicious cycle of metastatic cell growth and bone matrix degradation. Although this therapeutic strategy is plagued by numerous and severe side effects, such as musculoskeletal syndromes, recent research showed that targeting to a specific tissue, such as bone, and targeting specific MMPs which are overexpressed in the metastatic microenvironment, such as MMP-13, are viable strategies to minimize them.

Following both of these approaches, we demonstrated that a benzothiazole scaffold coupled with a bisphosphonic moiety is a versatile starting point for the development of bone-targeted MMP-13 inhibitors, with the possibility of tailoring their activity profile in order to also target other validated MMPs, mainly MMP-2, allowing for a mostly favorable therapeutic profile for the treatment of bone malignancies.

## Figures and Tables

**Table 1:** Compounds **1**–**12**.

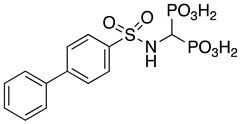	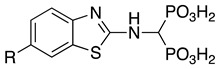
**ML115**	**1:** R = H **2:** R = F **3:** R = Cl **4:** R = NO_2_
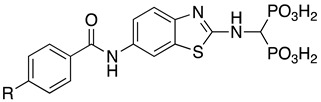	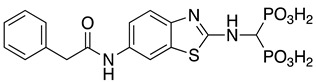
**5:** R = H **6:** R = Br **7:** R = NO_2_	**8**
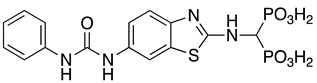	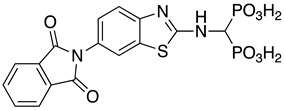
**9**	**10**
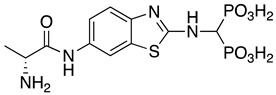	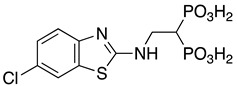
**11**	**12**

**Table 2:**
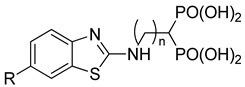
In vitro inhibitory activity was evaluated by fluorometric assay using commercially available catalytic domains of MMP-2, -8, -9, and -13. IC_50_ µM values are reported as the mean ± SEM (standard error of the mean) of at least 3 independent experiments, which were performed in triplicate.

*Compound*	*R*	*n*	*MMP-2*	*MMP-8*	*MMP-9*	*MMP-13*
ML 115			0.14 ± 0.04 ^a^	0.40 ± 0.03 ^a^	>100 ^a^	0.6 ± 0.3
1	H	0	12.7 ± 0.8	84.3 ± 2.1	>100	6.5 ± 1.2
2	F	0	9.0 ± 0.9	32.1 ± 1.9	26.5 ± 2.7	4.25 ± 0.35
3	Cl	0	2.64 ± 0.04	14.6 ± 0.2	18.7 ± 2.9	1.23 ± 0.12
4	NO_2_	0	4.5 ± 0.5	9.0 ± 0.4	7.7 ± 1.1	2.25 ± 0.15
5	NHCOPh	0	1.16 ± 0.18	9.0 ± 0.3	6.3 ± 0.9	0.670 ± 0.025
6	NHCOPh-4-Br	0	13.8 ± 0.9	60 ± 2.6	>100	6.7 ± 0.5
7	NHCOPh-4-NO_2_	0	0.98 ± 0.16	6.7 ± 0.8	5.4 ± 1.1	0.50 ± 0.01
8	NHCOCH_2_Ph	0	2.4 ± 0.5	10.8 ± 3.1	22 ± 8	9.3 ± 0.7
9	NHCONHPh	0	3.0 ± 0.4	15 ± 5	13.6 ± 1.4	11.5 ± 1.9
10	N(CO)_2_Ph	0	1.69 ± 0.08	12.0 ± 0.4	5.4 ± 1.6	10.9 ± 2.4
11	(*S*)-NHCOCH(CH_3_)NH_2_	0	9.4 ± 2.0	54 ± 6	>100	61.9 ± 1.3
12	Cl	1	4.7 ± 1.1	21.3 ± 1.7	36.6 ± 1.8	0.82 ± 0.06

^a^ Inhibition values from [19].

**Table 3:** Selectivity and ligand efficiency calculated on the basis of the IC_50_ values of Table 2.

Compound	Selectivity			Ligand Efficiency			
	*MMP-2*/*13*	*MMP-8*/*13*	*MMP-9*/*13*	*MMP-2*	*MMP-8*	*MMP-9*	*MMP-13*
*ML115*	0.23	0.67	>166.67	0.38	0.35	-	0.34
1	1.95	12.97	>15.4	0.36	0.30	-	0.38
2	2.12	7.55	6.24	0.35	0.31	0.31	0.37
3	2.15	11.87	15.20	0.38	0.33	0.32	0.41
4	2.00	4.00	3.42	0.33	0.31	0.32	0.35
5	1.73	13.43	9.40	0.29	0.25	0.25	0.30
6	2.06	8.96	>14.92	0.22	0.19	-	0.24
7	1.96	13.40	10.80	0.27	0.23	0.23	0.28
8	0.26	1.16	2.37	0.27	0.23	0.22	0.24
9	0.26	1.30	1.18	0.26	0.23	0.23	0.23
10	0.16	1.10	0.50	0.26	0.23	0.24	0.23
11	0.15	0.87	>1.62	0.28	0.23	-	0.23
12	5.73	25.98	44.63	0.35	0.31	0.29	0.40

## Data Availability

Data is contained in the article.

## Supplementary Materials

The following are available online at https://www.mdpi.com/1424-8247/14/2/85/s1: Figure S1. Docked pose of compound **7** (stick) in the MMP-13 binding site (grey cartoon) obtained with Glide (green C atoms) and Autodock (cyan C atoms).
