# Supplementary material for: (2-Aminobenzothiazole)-Methyl-1,1-Bisphosphonic Acids: Targeting Matrix Metalloproteinase 13 Inhibition to the Bone
Source: Pharmaceuticals (Basel). 2021 Jan 24;14(2):85. doi: 10.3390/ph14020085 (PMC7912614; doi:10.3390/ph14020085)
Supplement: Supplementary file 1 [file pharmaceuticals-14-00085-s001.pdf]

Supplementary material

(2-aminobenzothiazole)-methyl-1,1-bisphosphonic acids: targeting Matrix Metalloproteinase 13 inhibition to the bone.

Antonio Laghezza[1], Luca Piemontese[1], Leonardo Brunetti[1], Alessia Caradonna[1], Mariangela Agamennone[2], Fulvio Loiodice[1,*], Paolo Tortorella[1,*]

[1] Department of Pharmacy and Pharmaceutical Sciences, University of Bari "A. Moro", via E. Orabona 4, 70125 Bari, Italy; antonio.laghezza@uniba.it (A.L.); luca.piemontese@uniba.it (L.P.); leonardo.brunetti@uniba.it (L.B.); a.caradonna@hotmail.it (A.C.)

[2] Department of Pharmacy, University "G. d'Annunzio" of Chieti-Pescara, Via Dei Vestini, 31, 66100 Chieti, Italy; magamennone@unich.it (M.A.)

* Correspondence: paolo.tortorella@uniba.it (P.T.); fulvio.loiodice@uniba.it (F.L.)

Docking calculations with Autodock 4.2[1]

Methods

The MMP structures, previously aligned and prepared in Maestro with the Protein Preparation wizard as already described [2], were used as the macromolecules in the ADT interface [1]. The grid box was generated for each MMP to include the active site of the enzymes. A grid box of dimension X=32, Y=44, Z=48 was centered at the coordinates: x=66; y=26 and z=29, with a spacing of 0.375. Affinity maps were generated using AutoGrid4 and the atom types were extracted from ligand PDBQT file.

Docking calculations were carried out for ligands using a Lamarckian genetic algorithm and setting the maximum number of energy determination to the medium level. Docked geometries with highest calculated binding energy were analyzed and compared to those obtained with Glide.

Results

Generally, the docked poses obtained with Autodock are comparable to those obtained using Glide and described in the main text. Principal interactions concerning the zinc binding with one phosphonic group and occupation of the S1′ site with the long aromatic portion are confirmed. Moreover, Autodock produced poses showing both geometries observed also in Glide for the bisphosphonic group: with the second phosphonate lining up with the crystallographic geometry (such as the pose showed in Figure S1) or reaching Leu164 and Ala165 NH.

A comparison between the best docked pose of compound **7** in the MMP-13 active site obtained with Glide and Autodock is reported in Figure S1, as representative of the whole set of ligands.

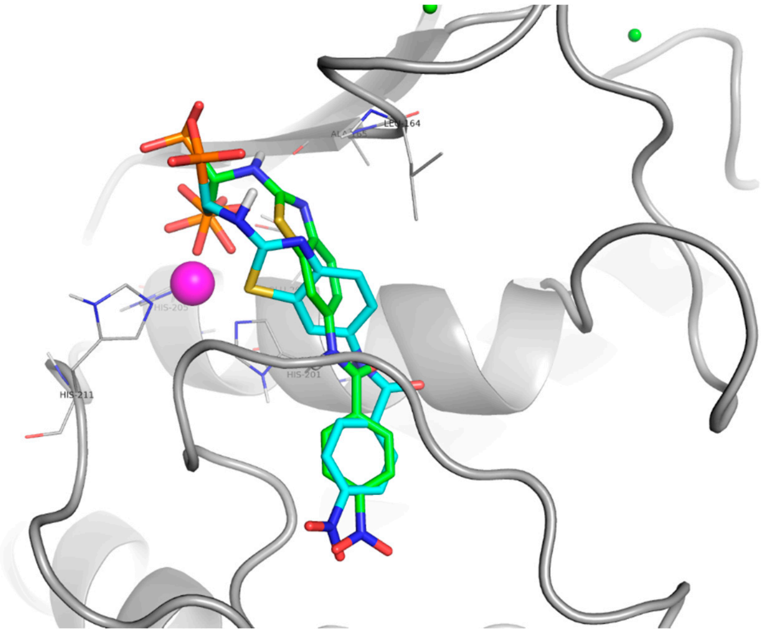

Figure S1. Docked pose of compound **7** (stick) in the MMP-13 binding site (grey cartoon). obtained with Glide (green C atoms) and Autodock (cyan C atoms).

References

[1] Morris, G. M., Huey, R., Lindstrom, W., Sanner, M. F., Belew, R. K., Goodsell, D. S. and Olson, A. J. Autodock4 and AutoDockTools4: automated docking with selective receptor flexiblity. J. Computational Chemistry 2009, 16: 2785-91. https://doi.org/10.1002/jcc.21256.

[2] Laghezza, A.; Piemontese, L.; Brunetti, L.; Caradonna, A.; Agamennone, M.; Di Pizio, A.; Pochetti, G.; Montanari, R.; Capelli, D.; Tauro, M.; Loiodice, F.; Tortorella, P. Bone-Seeking Matrix Metalloproteinase Inhibitors for the Treatment of Skeletal Malignancy. Pharmaceuticals 2020, 13 (6). https://doi.org/10.3390/ph13060113.
